# Circulating Chemokines and Short- and Long-Term Outcomes After Ischemic Stroke

**DOI:** 10.1007/s12035-024-04279-1

**Published:** 2024-06-11

**Authors:** Elzbieta Klimiec-Moskal, Piotr Koceniak, Kazimierz Weglarczyk, Agnieszka Slowik, Maciej Siedlar, Tomasz Dziedzic

**Affiliations:** 1 Department of Neurology, Jagiellonian University Medical College, Ul. Botaniczna 3, 31-503 Kraków, Poland; 2 Department of Clinical Immunology, Institute of Pediatrics, Jagiellonian University Medical College, Kraków, Poland

**Keywords:** Chemokines, Delirium, Inflammation, Outcome, Stroke

## Abstract

Chemokines are vital in post-cerebral ischemia inflammatory reactions. We investigate the possible relationship between plasma chemokines and short-term and long-term outcomes after stroke. This study included 235 patients (median age, 72 years; 49.8% female) suffering from ischemic stroke, or transient ischemic attack admitted to the hospital within 24 h of onset. We evaluated chemokines CCL2, CCL5, CXCL8, CXCL9, and CXCL10 in plasma samples collected upon admission. Further, we assessed functional outcomes at 3- and 12-months, all-cause fatality over 5 years, and episodes of delirium within the first 7 days of admission. Multivariate analysis revealed an association between higher CXCL10 levels and an increased risk of poor functional outcomes at 3 months (OR: 3.02, 95%CI: 1.22–7.46, *p* = 0.016) and 12 months (OR: 2.32, 95%CI: 1.03–5.26, *p* = 0.043), as well as an increased death risk (HR: 1.79, 95%CI: 1.04–3.07, *p* = 0.036). High CXCL8 levels independently predicted poor functional outcomes at 12 months (OR: 2.69, 95%CI: 1.39–6.31, *p* = 0.005) and a higher 5-year case fatality rate (HR: 1.90, 95%CI: 1.23–2.93, *p* = 0.004). Elevated CXCL9 levels also predicted unfavourable functional outcomes at 12 months (OR: 2.45, 95%CI: 1.07–5.61, *p* = 0.034). In univariate analysis, increased levels of CXCL8, CXCL9, and CXCL10 showed an association with delirium, although this link was not evident in the multivariate analysis. Plasma CXCL8 and CXCL10 show potential as prognostic biomarkers for stroke outcomes and as therapeutic targets suitable for reverse translation.

## Introduction

Chemokines are a family of small proteins that are secreted and play essential roles in immune cell migration into inflammatory sites [1, 2]. They are classified into four families: C, CC, CXC, and CX3C, based on the number of amino acids between the initial two cysteines.

Postmortem studies on animals and humans have demonstrated that cerebral ischemia initiates the expression of various chemokine classes in the brain, such as CCL2, CCL3, CCL5, CXCL8, CXCL10, and CXCL12 [3, 4]. These molecules are primarily produced by microglia and infiltrating immune cells. They regulate numerous biological processes like cell recruitment, blood–brain barrier permeability, angiogenesis, and neurogenesis. Depending on the timing and pathological conditions, chemokines and their receptors can have both positive and negative effects after a stroke [3, 4]. CCL2 and CCL5 are amongst chemokines that have a dual function in the pathophysiology of cerebral ischemia. On one hand, early after an ischemic stroke, CCL2 and CCL5 can exacerbate cerebral injury by facilitating the breakdown of the blood–brain barrier and promoting leukocyte infiltration in the brain [5–7]. Conversely, CCL2 may aid stroke recovery by attracting neural progenitor cells to the brain’s damaged regions [8], whilst CCL5 can safeguard neurons in per-infarct regions through the production of neurotrophic factors [9].

Moreover, chemokines and their receptors could serve as potential therapeutic targets post-stroke. For instance, inhibiting CXCL8 [10] or CCR5 [11] signalling has been observed to decrease brain damage and stimulate neurological repair in animal models of ischemic stroke. Additionally, the intravenous injection of a chemokine-binding protein that interacts with a wide range of chemokines reduced plasma chemokine levels, brain inflammation, and infarct size in mice [12].

Chemokines are released into the blood during cerebral ischemia. Both brain and peripheral organs, including lymph nodes and adipose tissue, are potential sources of circulating chemokines after stroke [13]. Elevated blood levels of CCL2 [14], CCL5 [15, 16], CXCL1 [16], CXCL5 [16], CXCL8 [17, 18]CXCL9 [19], and CXCL12 [19] have been reported in patients with acute ischemic stroke. In addition, multiple studies have reported an association between circulating chemokines and outcomes after stroke. Higher blood levels of CXCL12 [20], CXCL16 [21], and CCL23 [22] and lower levels of CCL11 [23] were associated with poor functional outcome and/or death.

However, the prognostic value of other chemokines, such as CCL2, CCL5, or CXCL8, which have been studied in animal stroke models, remains unclear. Considering experimental studies hinting at a dual role for chemokines during brain ischemia, further inquiry into their prognostic value in clinical contexts is warranted. Additionally, exploring the relationship between chemokines in the bloodstream and outcomes in stroke patients can help identify potential therapeutic targets worth investigating in reverse translation studies.

Here, we examine the potential correlation between specific plasma chemokines measured during the acute phase of an ischemic stroke and various short-term and long-term outcomes.

## Materials and Methods

This study’s participants were chosen from the patients involved in the Prospective Observational Polish Study on Delirium (PROPOLIS). PROPOLIS was a single-centre, prospective study conducted at the Department of Neurology, University Hospital, in Kraków, Poland [24]. Its main goal was to identify the incidence, predictors, and consequences of post-stroke delirium. The study included adults (18 years or older) who were admitted to the hospital within 48 h of having an acute stroke. Patients in a coma, experiencing alcohol withdrawal syndrome, affected by cerebral venous thrombosis or vasculitis, or those with a life expectancy less than 1 year were excluded. The study protocol (KBET/63/B/2014) was approved by the Bioethics Committee of Jagiellonian University, and each participant or their legal guardian provided informed consent. Patients who agreed to participate in PROPOLIS could opt out of blood sampling.

In this sub-study, we included patients who met specific criteria: (1) diagnosis of ischemic stroke or transient ischemic attack (TIA); (2) hospital admission within 24 h of symptom onset; and (3) consent given by the informed patient.

This study’s outcomes included unfavourable functional outcomes at both 3- and 12-month post-stroke, as defined by a modified Rankin Scale score exceeding 2. The study also looked at the 5-year all-cause fatality rate and the occurrence of delirium within the first week of hospital admission. Delirium was selected as a surrogate for short-term cognitive outcomes; this is justified by an increasing body of evidence suggesting chemokines are crucially involved in the pathogenesis of cognitive impairment [25, 26].

During the initial 7 days following admission, we conducted daily examinations for delirium symptoms. We employed the Brief Confusion Assessment Method (bCAM) [27] or the Confusion Assessment Method for Intensive Care Unit Patients (CAM-ICU) [28] and sourced additional information from the patient behaviour and cognitive fluctuation questionnaires completed by nurses. The final diagnosis of delirium was based on the criteria listed in the Diagnostic and Statistical Manual of Mental Disorders (DSM-5).

On admission, we evaluated neurological deficits using the National Institute of Health Stroke Scale (NIHSS). Pre-stroke dependency was determined by a score of 3–5 on the mRS scale. Five-year all-cause fatality data were procured from a government-maintained database.

Upon admission, venous blood was collected in heparinised tubes (Sarstedt, Germany), left at room temperature for 30 min, centrifuged, and then stored at -80 °C. We used a cytometric bead array immunoassay to measure CCL2, CCL5, CXCL8, CXCL9, and CXCL10 (Human Chemokine Kit, BD Bioscience, San Diego, CA, USA). Of the patients, 26.0% (61 patients) had CXCL-8 levels below the detection limit. In these instances, we substituted CXCL-8 concentrations with half of the lower limit of quantification.

We compared baseline characteristics and chemokine levels between groups using the χ2 test for proportions and the Mann–Whitney *U* test for continuous variables. Univariate logistic regression helped assess the associations between chemokines, functional outcomes, and delirium. Cox’s proportional hazard models were applied to analyse the death risk. For each chemokine, multivariate analyses adjusted for clinical predictors with a *p*-value less than 0.10 in the univariate models. We utilised receiver operating characteristic (ROC) curves and the Youden Index to identify chemokines’ optimal cut-off points to predict functional outcomes or delirium, balancing sensitivity and specificity. For survival analysis, the optimal cut-off points of chemokines were calculated using maximally selected rank statistics. All statistical tests were two-sided. A *p*-value of 0.05 was the threshold for statistical significance. The “Survminer” package (version 0.4.9) in the R statistical software was used for maximally selected rank statistics, whilst all other statistical analyses were carried out using STATA version 16.

## Results

A total of 574 patients met the inclusion criteria, with a median age of 73.5 years (interquartile range [IQR]: 64–82), median NIHSS score of 6 (IQR: 3–15), and 53.1% were female. Amongst these, blood samples were available for 235 patients, with a median age of 72 years (IQR: 63–82), a median NIHSS score of 5 (IQR: 2–11), and 49.8% were female. Out of these, 211 patients (89.8%) had suffered a stroke, and 24 (10.2%) had TIA. No blood samples were available from the remaining 339 patients (median age: 74 years, IQR: 64–82; median NIHSS: 8, IQR: 3–16; 55.8% female), either because they declined blood sampling or because their blood was used in other experiments. Compared to the patients included in the study, those for whom blood samples were not available had a higher admission NIHSS score (*p* < 0.001), but their ages (*p* = 0.364) and genders (*p* = 0.159) were similar.

We had data on delirium and all-cause fatality over 5 years for all patients included in the study. However, we had missing data on functional outcomes at 3 and 12 months for 19 (8.1%) and 16 (6.8%) patients, respectively.

### 3-Month Functional Outcome

The baseline characteristics of the patients are displayed in Table 1. Of these, 99 patients (accounting for 45.8%) experienced poor functional outcomes 3 months post-stroke.

**Table 1:** Comparison between good outcome and poor outcome group 3 and 12 months after stroke

	Good 3-month outcome *N* = 117	Poor 3-month outcome *N* = 99	*p*-value	Good 12-month outcome *N* = 118	Poor 12-month outcome *N* = 101	*p*-value
Age, median (IQs)	67 (58–77)	79 (67–85)	< 0.001	65 (57–74)	80 (73–86)	< 0.001
Female, *n* (%)	55 (47.0)	52 (52.5)	0.419	58 (49.2)	53 (52.5)	0.624
Hypertension, *n* (%)	80 (68.4)	76 (76.8)	0.170	77 (65.3)	80 (79.2)	0.022
Diabetes mellitus, *n* (%)	27 (23.1)	29 (29.3)	0.299	29 (24.6)	31 (30.7)	0.312
Atrial fibrillation, *n* (%)	14 (12.0)	32 (32.3)	< 0.001	13 (11.0)	34 (33.7)	< 0.001
Myocardial infarction, *n* (%)	19 (16.2)	14 (14.1)	0.669	18 (15.3)	14 (13.9)	0.771
Previous stroke or TIA, *n* (%)	18 (15.4)	20 (20.2)	0.351	19 (16.1)	20 (19.8)	0.472
Pre-stroke dependency, *n* (%)	3 (2.6)	38 (38.4)	< 0.001	4 (3.4)	39 (38.6)	< 0.001
NIHSS score on admission, median (IQs)	3 (1–5)	10 (5–18)	< 0.001	3 (1–6)	9 (4–18)	< 0.001
Stroke location, *n* (%)			0.420			0.089
Right hemisphere	40 (34.2)	44 (44.4)		39 (33.1)	46 (45.5)	
Left hemisphere	57 (48.7)	40 (40.4)		55 (46.6)	44 (43.6)	
Posterior fossa	18 (15.4)	12 (12.1)		22 (18.6)	8 (7.9)	
Multiple locations	2 (1.7)	3 (3.0)		2 (1.7)	3 (3.0)	
Reperfusion therapy (thrombolysis or/and thrombectomy), *n* (%)	34 (29.1)	35 (35.4)	0.323	35 (29.7)	30 (29.7)	0.995

**Fig. 1:**
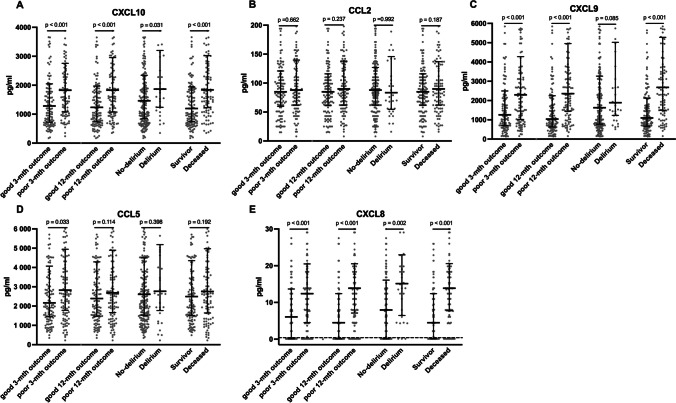
Chemokine levels stratified by outcomes. Each panel displays a scatter plot of the respective chemokine levels grouped by outcomes: **A** CXCL10, **B** CCL2, **C** CXCL9, **D** CCL5, **E** CXCL8. Error bars represent median levels with interquartile ranges. In panel **E**, data points below the dotted line correspond to CXCL8 values that fell below the quantification limit and were arbitrarily imputed as half of the lower limit of quantification

**Fig. 2:**
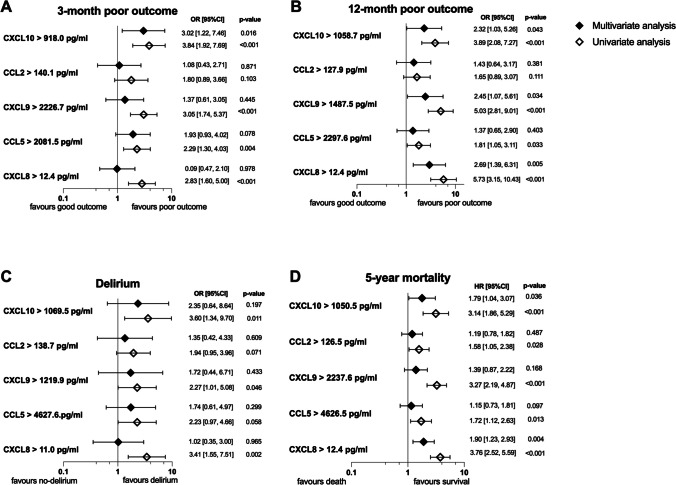
Results for uni- and multivariate analyses

Patients with worse outcomes exhibited heightened levels of CXCL10 (median: 1826 vs 1284.4 pg/ml, *p* < 0.001), CXCL9 (2346.9 vs 1258.7 pg/ml, *p* < 0.001), CCL5 (2864.4 vs 2165.2 pg/ml, *p* = 0.033), and CXCL8 (12.4 vs 6.1 pg/ml, *p* < 0.001) (Fig. 1). These elevated cytokine levels were associated with negative outcomes in the univariate analysis. However, when adjusting for factors like age, atrial fibrillation, NIHSS score on admission, and pre-stroke dependency in the multivariate analysis, only CXCL10 remained as an independent outcome predictor (Fig. 2).

### 12-Month Functional Outcome

One hundred and one patients (accounted for 46.1%) demonstrated a poor functional outcome 12 months post-stroke. They were characterised by elevated plasma levels of CXCL10 (1853 vs 1235.9 pg/ml), CXCL9 (2677.2 vs 1038.5 pg/ml), and CXCL8 (13.9 vs 4.5 pg/ml), all with a *p*-value of less than 0.001. In a univariate analysis, poor outcomes were associated with elevated levels of CXCL10, CXCL9, CCL5, and CXCL8. However, after adjusting for age, hypertension, atrial fibrillation, NIHSS admission score, right-sided lesion, and pre-stroke dependency in a multivariate analysis, only higher levels of CXCL10, CXCL9, and CXCL8 remained independent predictors of outcome.

### All-Cause 5-Year Case Fatality

Over 5 years following a stroke, 104 patients (44.3%) passed away. These patients exhibited higher levels of CXCL10 (1841.9 vs 1198.5 pg/ml, *p* < 0.001), CXCL9 (2683.7 vs 1095.7 pg/ml, *p* < 0.001), and CXCL8 (13.9 vs 4.5 pg/ml, *p* < 0.001). Univariate analysis revealed an association between elevated chemokine levels and an increased risk of death. In a multivariate analysis adjusted for age, atrial fibrillation, pre-stroke dependency, NIHSS on admission and right-sided lesion, CXCL10 and CXCL8 still remained independent predictors of death (Table 2).

**Table 2:** Comparison between no-delirium vs. delirium group and between survivors vs. deceased patients

	No-delirium *N* = 201	Delirium *N* = 34	*p*-value	Survivors *N* = 131	Deceased *N* = 104	*p*-value
Age, median (IQs)	70 (61–80)	82 (71–85)	0.003	65 (58–75)	80 (72.5–86)	< 0.001
Female, *n* (%)	97 (48.3)	20 (58.8)	0.255	67 (51.1)	50 (48.1)	0.640
Hypertension, *n* (%)	141 (70.1)	25 (73.5)	0.689	88 (67.2)	78 (75.0)	0.191
Diabetes mellitus, *n* (%)	46 (22.9)	14 (41.2)	0.024	28 (21.4)	32 (30.8)	0.101
Atrial fibrillation, *n* (%)	32 (15.9)	16 (47.1)	< 0.001	15 (11.5)	33 (31.7)	< 0.001
Myocardial infarction, *n* (%)	30 (14.9)	4 (11.8)	0.628	18 (13.7)	16(15.4)	0.772
Previous stroke or TIA, *n* (%)	35 (17.4)	5 (14.7)	0.680	22 (16.8)	18 (17.3)	0.912
Pre-stroke dependency, *n* (%)	26 (12.9)	19 (55.9)	< 0.001	11 (8.4)	34 (32.7)	< 0.001
NIHSS score on admission, median (IQs)	4 (2–8)	17 (12–20)	< 0.001	3 (1–6)	8 (3.5–18)	< 0.001
Stroke location, *n* (%)			0.078			0.020
Right hemisphere	69 (34.3)	20 (58.8)		42 (32.1)	47 (45.2)	
Left hemisphere	95 (47.3)	12 (35.3)		60 (45.8)	47 (45.2)	
Posterior fossa	32 (15.9)	2 (5.9)		27 (20.6)	7 (6.7)	
Multiple locations	5 (2.5)	0 (0.0)		2 (1.5)	3 (2.9)	
Reperfusion therapy (thrombolysis or/and thrombectomy), *n* (%)	53 (26.4)	20 (58.8)	< 0.001	43 (32.8)	30 (28.8)	0.513

### Delirium

Delirium was observed in 34 patients, comprising 14.5% of the total. These patients showed higher levels of CXCL10 (1868.3 vs 1465.3 pg/ml, *p* = 0.031) and CXCL8 (15.1 vs 8.0 pg/ml, *p* = 0.002). Univariate analysis revealed that elevated levels of CXCL10, CXCL9, and CXCL8 were linked to an increased delirium risk. However, a multivariate analysis adjusting for age, diabetes mellitus, atrial fibrillation, pre-stroke dependency, NIHSS admission score, the presence of a right-sided lesion, and reperfusion therapy found that these chemokines could not predict delirium occurrence.

## Discussion

We found that higher CXCL10 plasma levels correlate with unfavourable immediate and long-term functional outcomes and a higher fatality rate within 5 years of an ischemic stroke. Additionally, an elevation in CXCL8 blood levels is associated with a greater risk of poor functional outcomes after a year and a higher likelihood of death within 5 years of experiencing a stroke. Furthermore, higher levels of CXCL9 in plasma have been found to predict a poor functional outcome after 1 year.

CXCL10, also known as interferon gamma-induced protein 10, functions as a chemoattractant for monocytes, macrophages, T cells, and natural killer (NK) cells [29]. Animal studies have demonstrated an increased expression of CXCL10 and its receptor, CXCR3, in the ischemic brain [30–32]. This is supported by human postmortem analyses, revealing up-regulation of CXCL10 in the brains of ischemic stroke patients [33]. It has also been observed that CXCL10 might exacerbate blood–brain barrier damage caused by NK cells during cerebral ischemia [32]. Interestingly, patients with higher blood levels of CXCL10 have been associated with a worse prognosis following intracerebral haemorrhage [34].

Our study found that plasma CXCL8 (interleukin-8) is linked with long-term outcomes post-stroke. In rabbits exposed to transient cerebral ischemia, CXCL8 levels increased 6 h after reperfusion [10]. CXCL8 is a strong neutrophil chemoattractant, which has an important role in the post-ischemic brain [35]. The reduction of infarct size was observed after either silencing the CXCL8 gene or applying a neutralising anti-CXCL8 antibody [10, 36]. Similarly, using reparixin, a CXCL8 receptor blocker, improved long-term neurological recovery in a cerebral ischemia rat model [37]. In acute ischemic stroke patients, a correlation was found between serum CXCL8, infarct volume, and mRS assessed at 90 days [38].

We discovered that elevated blood levels of CXCL9 indicated a potentially unfavourable functional outcome after 12 months. CXCL9, a chemoattractant for T lymphocytes and NK cells triggered by interferon-gamma [1], is still not clearly understood in the context of cerebral ischemia. However, one study found a correlation between higher serum CXCL9 levels and increased neurological deficits and infarct volume [19].

Delirium is a serious neuropsychiatric syndrome that causes sudden changes in awareness, attention, and cognition [39]. Increased blood levels of CXCL8 have been noted in elderly patients with delirium [40]. However, studies involving critically ill patients have yielded inconsistent results regarding blood CXCL8 levels in delirious patients [41, 42]. Some research has indicated a potential connection between blood CCL2 and delirium [42, 43]. The link between circulating chemokines and post-stroke delirium has not yet been studied. In our research, we found higher levels of CXCL8, CXCL9, and CXCL10 to be associated with delirium in univariate analysis. However, this association disappeared when adjusted for clinical predictors. We must acknowledge the possibility that our sample size was insufficient to confirm a relationship between chemokines and delirium in multivariate analysis.

Our study has several limitations. Firstly, our limited sample size may impact the reliability of our findings. Secondly, selection bias may have occurred as patients without available blood samples were excluded. Thirdly, with 26% of patients having CXCL8 below the quantifiable limit, the precision of CXCL8 measurements and the rigour of statistical analyses might be compromised. Also, we only measured chemokine levels once. More reliable data about the relationship between chemokine kinetics and prognosis could be obtained with multiple measurements of blood chemokines. Lastly, our observation-based approach prevents us from establishing a causal link between chemokines and stroke outcomes.

In conclusion, plasma CXCL8 and CXCL10 show potential as both prognostic biomarkers for stroke outcomes and therapeutic targets. Further research on this topic, specifically using animal models, is warranted.

## Data Availability

The datasets used during the current study are available from the corresponding author on reasonable request.
